# Cross-cultural adaptation and validation of the VISA-A questionnaire for Chilean Spanish-speaking patients

**DOI:** 10.1186/s13018-018-0882-2

**Published:** 2018-07-13

**Authors:** Andres Keller, Pablo Wagner, Guillermo Izquierdo, Jorge Cabrolier, Nathaly Caicedo, Emilio Wagner, Nicola Maffulli

**Affiliations:** 1 Department of Orthopedics, Universidad del desarrollo - Clinica Alemana de Santiago, Vitacura 5951, 7650568 Santiago, Chile; 2 Universidad de los Andes - Hospital Militar de Santiago, Santiago, Chile; 3 Centre for Sports and Exercise Medicine, Barts and The London School of Medicine and Dentistry, Mile End Hospital, London, UK

**Keywords:** VISA-A, Achilles tendinopathy, Score validation, Spanish validation

## Abstract

**Background:**

The purpose of this study is to translate, culturally adapt, and validate the VISA-A questionnaire for Chilean Spanish speakers with Achilles tendinopathy (AT), which has been originally developed for English-speaking population.

**Methods:**

According to the guidelines published by Beaton et al., the questionnaire was translated and culturally adapted to Chilean patients in six steps: initial translation, synthesis of the translation, back translation, expert committee review, test of the pre-final version (cohort *n* = 35), and development of VISA-A-CH. The resulting Chilean version was tested for validity on 60 patients: 20 healthy individuals (group 1), 20 patients with a recently diagnosed AT (group 2), and 20 with a severe AT that already initiated conservative treatment with no clinical improvement (group 3). The questionnaire was completed three times by each participant: at the time of study enrollment, after an hour, and after a week of the initial test.

**Results:**

All six steps were successfully completed for the translation and cultural adaptation of the VISA-A-CH. VISA-A-CH final mean scores in the healthy group was significantly higher than those in the other groups. Group 3 had the lowest scores. Validity showed excellent test-retest reliability (rho *c* = 0.999; Pearson’s *r* = 1.000) within an hour and within a week (rho *c* = 0.837; Pearson’s *r* = 0.840).

**Conclusions:**

VISA-A was translated and validated to Chilean Spanish speakers successfully, being comparable to the original version. We believe that VISA-A-CH can be recommended as an important tool for clinical and research settings in Chilean and probably Latin-American Spanish speakers.

**Electronic supplementary material:**

The online version of this article (10.1186/s13018-018-0882-2) contains supplementary material, which is available to authorized users.

## Background

Achilles tendinopathy (AT) is the most common cause of posterior heel pain [1–3] in athletes and non-athletes [4]. The activities most related to this pathology are those involving jumping and running [4, 5]. The incidence of AT has been increasing in the last decades, with a prevalence of 10% in runners [4, 6]. In terms of its clinical presentation, pain in the middle portion of the Achilles tendon during and after physical activity, increased volume in the involved region of the tendon, and morning stiffness are frequent. The above symptoms usually decrease when the patient reduces the load or the level of activity but tend to recur when the activity is resumed [7, 8]. For these reasons, AT is a frequent cause of limitation in the physical activity of patients, with the consequent negative impact on their general health.

Despite the high incidence and its multi-factorial etiology [9], the decision to undertake conservative or operative treatment in patients with AT is still under debate [10], though surgical treatment is generally recommended only following failure of conservative measures [11, 12]. Regardless of the management path chosen, the aim of treatment is to return the patient to clinical and functional wellbeing. The achievement of these objectives can be difficult to compare objectively between different studies, mainly because of the ways in which they are measured, and possible difference in severity and clinical presentation of the condition.

A simple, self-administered questionnaire was developed by the Victorian Institute of Sports Assessment to be completed by patients with AT, called VISA-A (Victorian Institute of Sport Assessment-Achilles Questionnaire), which assesses several aspects such as: pain (questions 1–3), function (questions 4–6), and activity (questions 7 and 8). For this reason, it can be used to determine the clinical severity of the condition and provide a guide for treatment, as well as to monitor the effects of treatment, constituting a validated, reliable, and accurate tool to specifically evaluate patients with AT [13]. Moreover, it is useful to compare results between different studies [14–17]. The questionnaire was developed for English-speaking populations: this is the reason why it is necessary to translate, culturally adapt, and validate [18] the VISA-A in other languages. This has already been the case for the Swedish- [14], Italian- [16], and German-speaking populations [19].

The purpose of this study was to translate, culturally adapt, and validate the VISA-A questionnaire for the Spanish-speaking Chilean population with AT.

## Methods

According to the guidelines published by Beaton et al. [18], the questionnaire was translated and culturally adapted to Spanish-speaking Chilean patients in six steps.

Step 1. Initial translation: two bilingual translators whose mother tongue was Spanish developed two independent translations. One of the translators had medical knowledge of the concepts and terms (an orthopedic surgeon with specialization in foot and ankle); the other translator had no medical knowledge or relation with health care system (native translator).

Step 2. Synthesis of translation: both translators agreed on their translations and developed a common translation (preliminary translation V1.0).

Step 3. Retrograde translation: with the preliminary version V1.0 in hand and blind to the original version (VISA-A), two non-medically expert translators whose mother tongue was English translated the questionnaire back into English.

Step 4. Expert committee: a committee was organized with the participation of the original translators, other expert translators, orthopedic surgeons with experience in foot and ankle surgery, and other health care professionals, to review all translations and to develop the pre-final version of the questionnaire (V2.0).

Step 5. Test of the V2.0 questionnaire: this stage ensured that the adapted version was equivalent to the original version. The questionnaire was completed by a cohort of 35 people. Later, they were interviewed to discuss errors, what they understood of each question, and the difficulty to answer them.

Step 6. Development of the final version (VISA-A-CH): after analyzing the opinions, doubts, and suggestions of the interviewees, corrections were made to V2.0 in each one of its items, with the final version being developed V3.0 (VISA-A-CH) (Additional file 1).

After the translation and cultural adaptation, the VISA-A-CH questionnaire was subjected to validation.

After approval by the local ethics committee, the VISA-A-CH was prospectively delivered to 60 patients divided into three groups: group 1, 20 healthy patients with no AT symptoms and signs (control group); group 2, 20 patients with a recently diagnosed AT; and group 3, 20 patients with severe AT who already initiated conservative management with no clinical improvement. These patients were all older than 18, and all had been selected and evaluated by a trained foot and ankle orthopedic surgeon in a single center in Santiago, Chile. Informed consent was obtained from all individual participants included in the study.

The questionnaire was completed anonymously three times by each participant: at the time of study enrollment (time 0), after 1 h (time + 1), and after 1 week of the initial test (time + 7).

Descriptive statistics were calculated, using percentile, mean, and standard deviation. The questionnaires (three each participant, 180 in total) were evaluated using mixed models to control intra- and inter-patient variability. We used significance level of 95%, and the data were processed with software STATA version 14.0.

## Results

To develop the translation and cultural adaptation of the questionnaire (step 5 from the “Methods” section), initially, a cohort of 23 men and 12 women with age of 18 to 50 years were interviewed to check for score structure and reading comprehension. Half of them were fourth year medical students from a Chilean University. The other half were firefighters from the regional fire station. Each participant was given a copy of VISA-A-CH V2.0 to read and respond as directed. At the end of the survey, feedback on the questionnaire was requested.

**Fig. 1:**
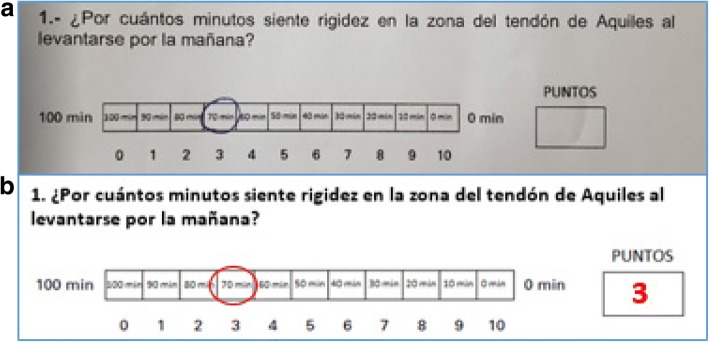
Example of incorrectly answered question during the test adaptation. **a** The participant did not fill the “PUNTOS” (score) box. **b** Correct way of answering

Twenty-seven participants (77%) did not answer the questionnaire in a structural correct form (i.e., ticking the relevant box) as shown in Fig. 1.

Regarding the comprehension of the questions, all the participants reported having no problem understanding what was being asked.

On the other hand, 16 participants (45%) reported that in some of the questions there was no precise alternative for someone healthy and asymptomatic (for example, the maximum score for question no. 7 was: “Yes, I do sport or physical activity to the same or even to a higher level since the discomfort began”). Healthy participants have never experienced any discomfort. However, as expected, they responded correctly with the maximum score for those questions.

For the validation stage of VISA-A-CH, three groups were selected to complete the questionnaire as explained in the “Methods” section: group 1: 20 healthy subjects, 10 men and 10 women, with a mean age of 38 years (range 20–55); group 2: 20 patients with a recently diagnosed AT, 13 men and 7 women, with a mean age of 41 years (25–49); and group 3: 20 patients with a severe AT that already initiated conservative treatment with no clinical improvement, 14 men and 6 women, with a mean age of 43 years (29–51).

**Fig. 2:**
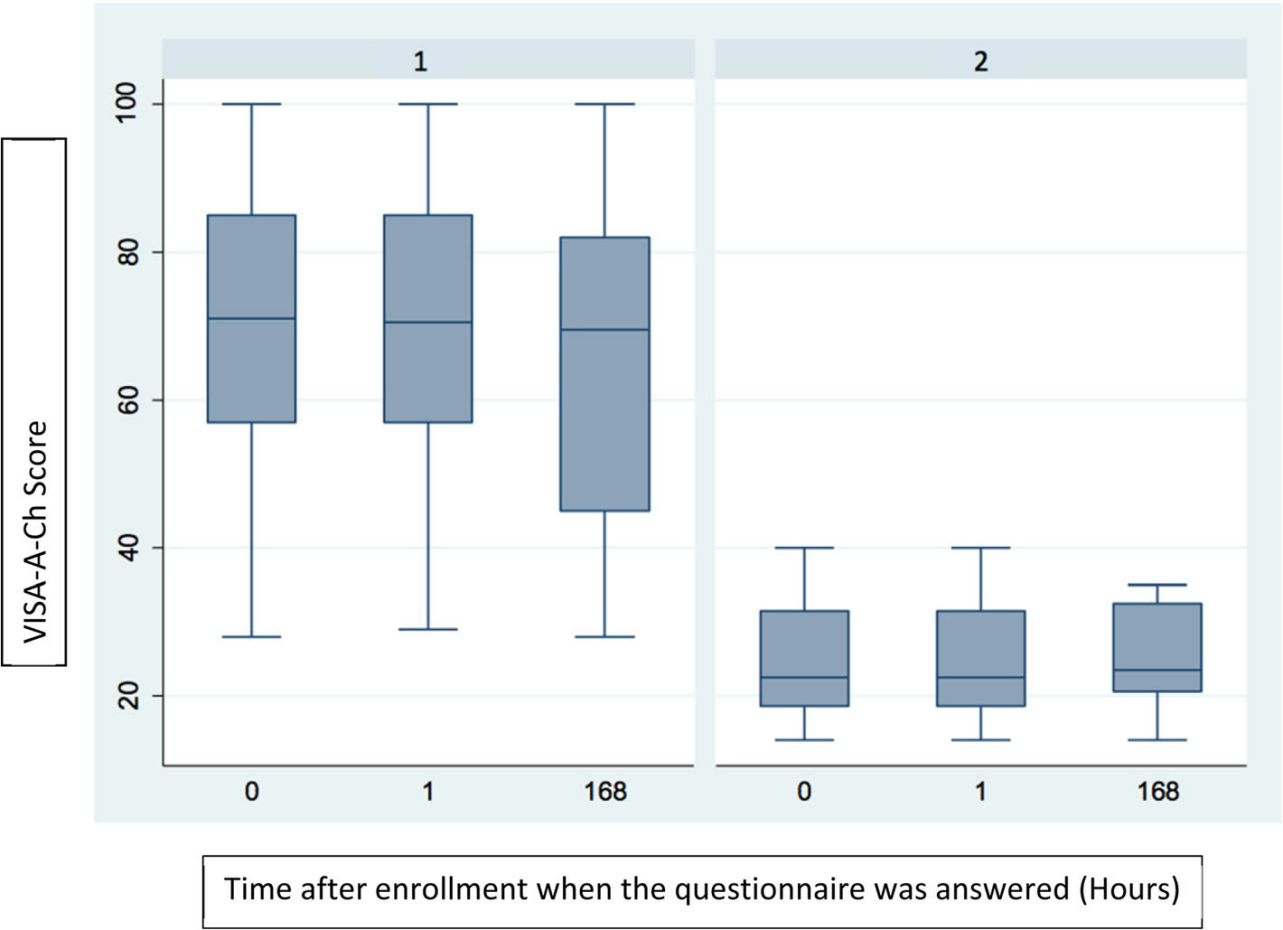
Figure showing scores (*y* axis) in groups 2 and 3 throughout time (*x* axis, in hours). 0, 1, and 168 h (7 days). Column 1, group 2; column 2, group 3

**Fig. 3:**
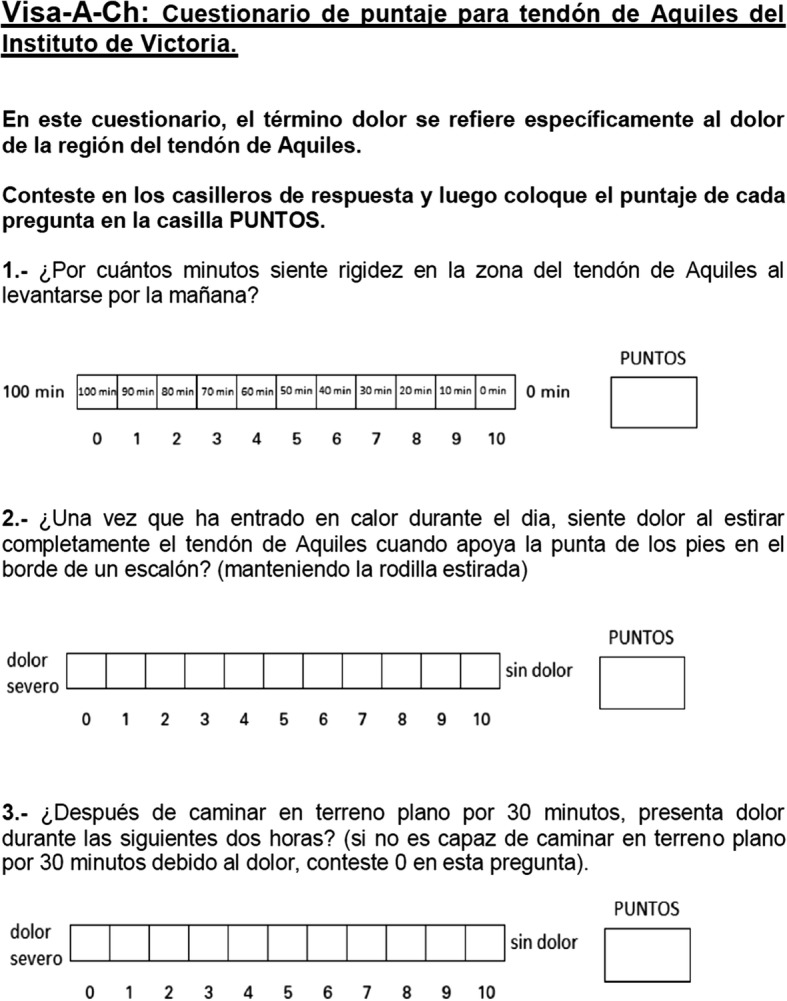
Image of the final version of the VISA-A-CH questionnaire

**Fig. 4:**
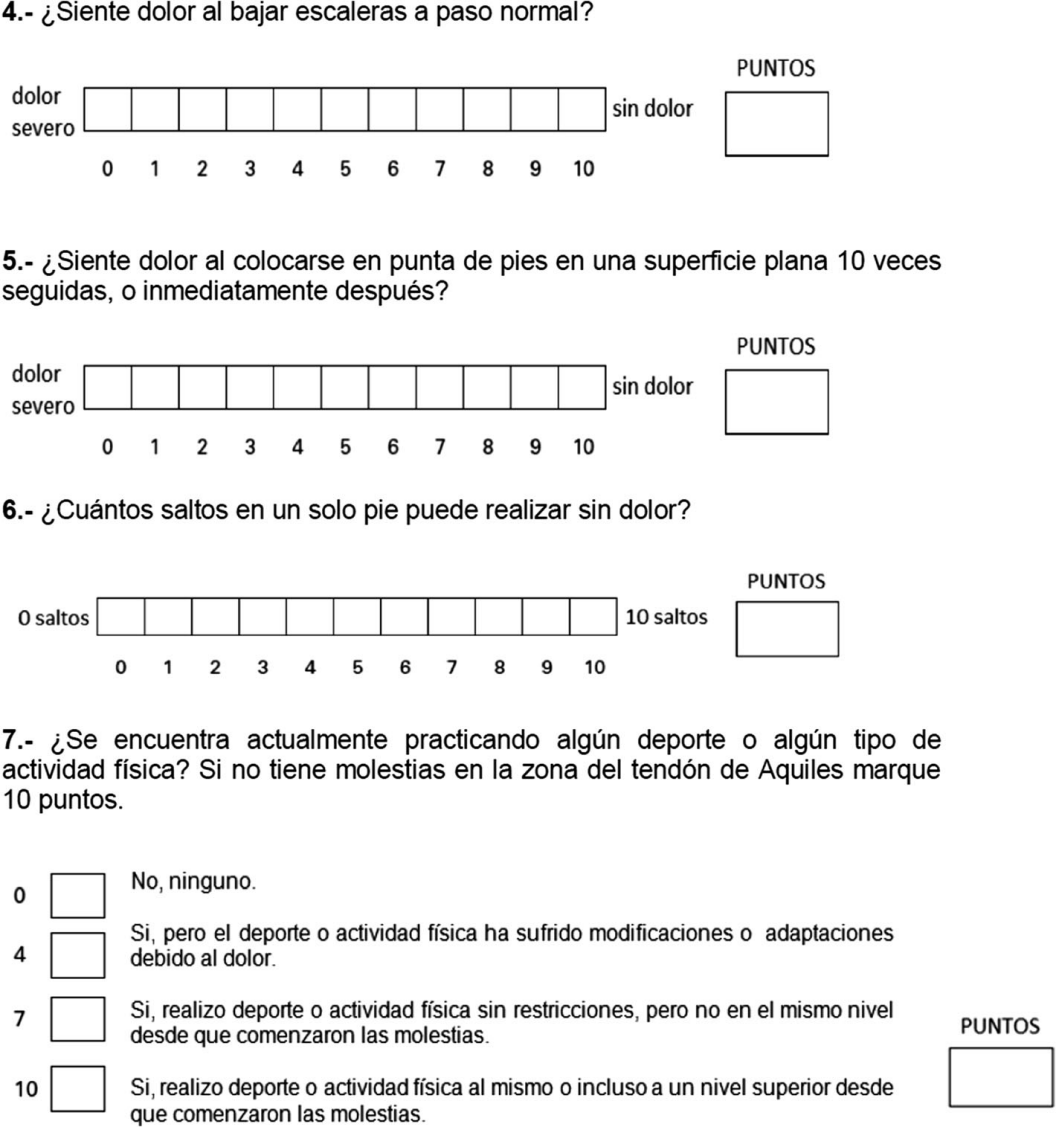
Image of the final version of the VISA-A-CH questionnaire

**Fig. 5:**
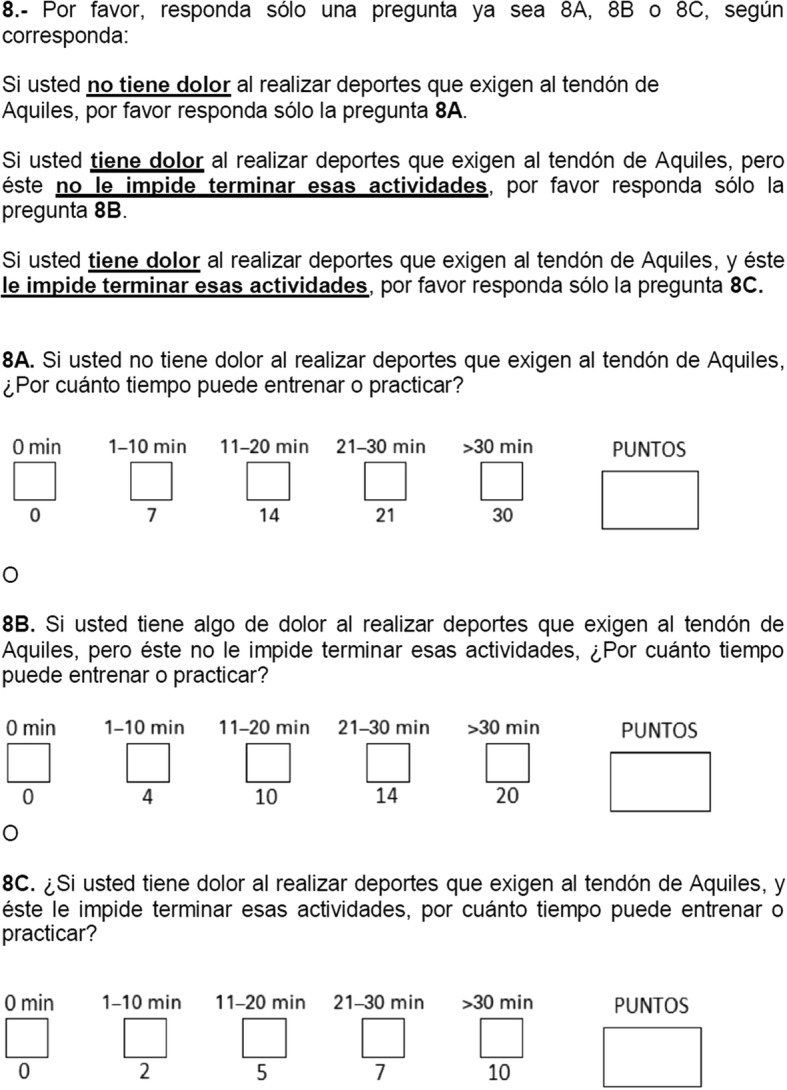
Image of the final version of the VISA-A-CH questionnaire

The control group obtained 100 points in all measurements at all time points (at the time of enrollment, after 1 h, and after a week). Group 3 had the lowest scores of all the groups at all time points (time 0, + 1, + 7). Tables 1 and 2 show the scores of groups 2 and 3 (Tables 1 and 2). When comparing questionnaire results in groups 2 and 3, the *p* value of 0.335 indicates that there was no significant change in re-test scores at an hour (rho *c* = 0.999; Pearson’s *r* = 1000) or at a week from diagnosis (rho *c* = 0.837; Pearson’s *r* = 0.840). Figure 2 depicts the scores obtained by the patients in groups 2 and 3 at different time points, showing no change. The final version of the VISA-A-CH questionnaire is shown in Figs. 3, 4, and 5.

**Table 1:** VISA-A-CH scores of patients with AT (group 2) at different time points

Answer time (hours)	*N*	Min	p50	Max	Mean
0	20	28	71	100	67.16
1	20	29	70.5	100	67.33
168	20	28	69.5	100	65.27

**Table 2:** VISA-A-CH scores of patients with AT and failure to conservative treatment (group 3)

Answer time (hours)	*N*	Min	p50	Max	Mean
0	20	14	22.5	40	24.7
1	20	14	22.5	40	24.7
168	20	14	23.5	35	26

## Discussion

With the multinational advance of research, it is necessary when comparing studies to have equivalent scoring systems, questionnaires, and results [20, 21]. In the case of self-administered questionnaires, comparing results between studies with different language and culture populations may lead to systematic errors if these assessment tools are not equivalent to the original [22]. Since most questionnaires are developed in English, they have to be validated in other languages. This is not just a translation issue: when the questionnaire is used in another country or with immigrants, it must necessarily be culturally adapted. Beaton et al. developed a clinical guideline for cultural translation and adaptation of self-report scores [18]. It has progressive stages of translation, synthesis, reverse translation, expert committee, test of the questionnaire, and development of the final version.

The VISA-A, originally developed in English, is a reliable and reproducible questionnaire to compare results among patients with different degrees of AT severity. We have to remember it is not a diagnostic tool, but a valid way to measure the condition of the Achilles tendon. Its continuous numerical score has the potential to be used in clinical and research settings, but it was not designed to be a diagnostic tool [13]. Although the translation of the VISA-A was not difficult, cultural adaptation in relation to how to complete the questionnaire was the first inconvenience we encountered. Although the score of these evaluations was not affected, it showed how unclear it was in its filling instructions (77% of respondents did not respond in the boxes requested). For this reason, the VISA-A-CH developers added an instruction phrase on how to fill it: “Answer in the answer boxes and then fill the box labeled PUNTOS (translation for score) with the score for each question.” When analyzing test-retest reliability, no significant changes were found between the scores obtained in all patient groups. They all had scores not statistically different at time 0, + 1 and + 7. A week after diagnosis, groups 2 and 3 patients, despite the medical indications and the schedule of eccentric exercises given to the patients, showed no change in score, perhaps highlighting that, despite the correct exercises having been prescribed and implemented, in the short term, the symptoms of AT do not change.

We point out that the use of the VISA-A-CH is likely to transcend the country where it was developed, namely Chile, and be used in all the Spanish-speaking countries in Latin America. We are aware of the linguistic differences which have developed during centuries between the Spanish language spoken in Spain and that of Latin America. In this respect, we suspect that a separate cross-cultural adaptation will be needed for Spain.

Regarding the limitations of the study, the number of participants in the different stages was established according to previous studies of cultural adaptation to other languages, without calculation of sample size. On the other hand, it should be noted that the patients and controls included in both the translation and validation stages are of the same socioeconomic level, not including patients with other education/cultural levels.

## Conclusions

In conclusion, the VISA-A questionnaire was successfully translated and culturally adapted to a Chilean Spanish-speaking populace, carefully following the published guidelines for this process. The ability to measure aspects such as pain and functionality in physical activity highlight the utility of VISA-A-CH for patients with AT. This type of studies is fundamental for subsequent clinical work without methodological errors that occur when patients are evaluated with questionnaires that have not been properly translated and culturally adapted.

## Additional file

Additional file 1: VISA-A-CH. (DOCX 227 kb)
