# Supplementary figures and images for: Cross-cultural adaptation and validation of the VISA-A questionnaire for Chilean Spanish-speaking patients
Source: J Orthop Surg Res. 2018 Jul 13;13:177. doi: 10.1186/s13018-018-0882-2 (PMC6045880; doi:10.1186/s13018-018-0882-2)

**Additional file 1. VISA-A-CH**


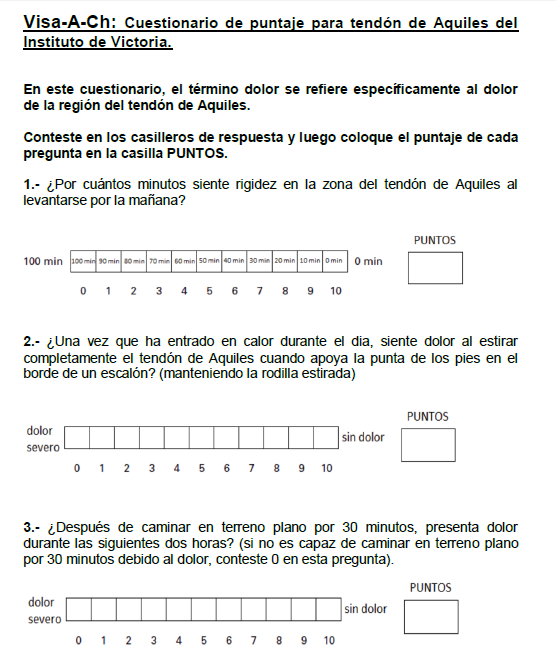


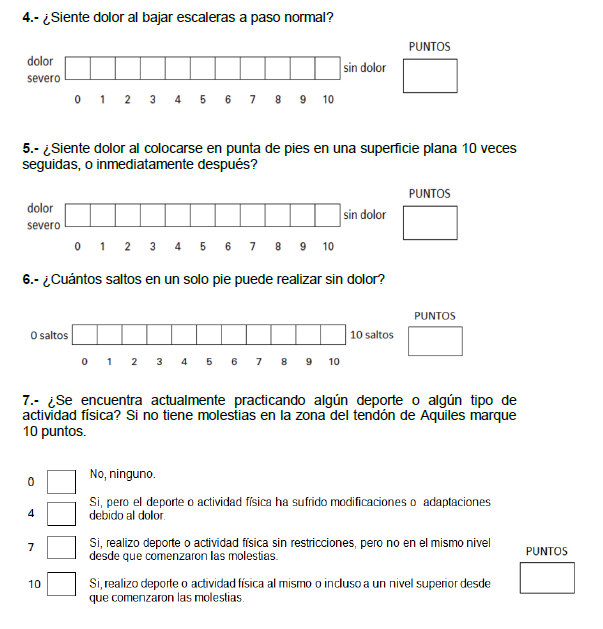


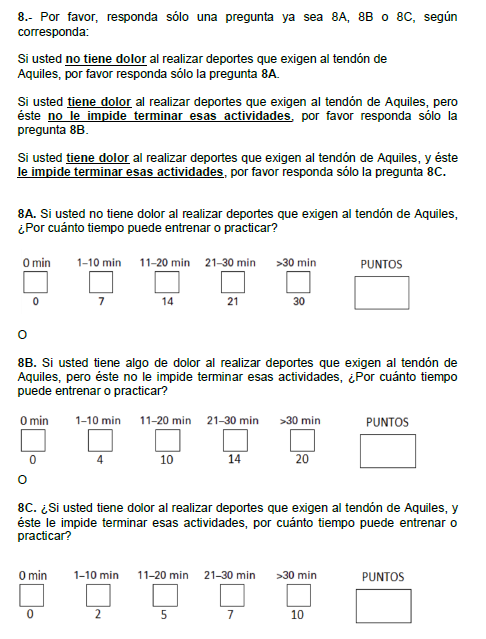

Supplement: Supplementary file 1 — VISA-A-CH. (DOCX 227 kb) [file 13018_2018_882_MOESM1_ESM.docx]
